# Fragmenstein: predicting protein–ligand structures of compounds derived from known crystallographic fragment hits using a strict conserved-binding–based methodology

**DOI:** 10.1186/s13321-025-00946-0

**Published:** 2025-01-13

**Authors:** Matteo P. Ferla, Rubén Sánchez-García, Rachael E. Skyner, Stefan Gahbauer, Jenny C. Taylor, Frank von Delft, Brian D. Marsden, Charlotte M. Deane

**Affiliations:** 1 Oxford Protein Informatics Group, Department of Statistics, University of Oxford, Oxford, UK; 2 Centre for Medicine Discoveries, Nuffield Department of Medicine, University of Oxford, Oxford, UK; 3 Wellcome Centre for Human Genetics, NIHR Oxford BRC Genomic Medicine, University of Oxford, Oxford, UK; 4 Diamond Light Source, Science and Technology Facilities Council, Oxford, UK; 5 OMass Therapeutics, ARC Oxford, Oxford, UK; 6 Department of Pharmaceutical Chemistry, University of California San Francisco, San Francisco, USA; 7 Department of Biochemistry, University of Johannesburg, Johannesburg, South Africa

**Keywords:** Fragment-based drug design, Hit discovery, De novo design, Fragment merging, Fragment linking, Fragment elaboration, Conformer generation, Molecular mechanics

## Abstract

Current strategies centred on either merging or linking initial hits from fragment-based drug design (FBDD) crystallographic screens generally do not fully leaverage 3D structural information. We show that an algorithmic approach (Fragmenstein) that ‘stitches’ the ligand atoms from this structural information together can provide more accurate and reliable predictions for protein–ligand complex conformation than general methods such as pharmacophore-constrained docking. This approach works under the assumption of conserved binding: when a larger molecule is designed containing the initial fragment hit, the common substructure between the two will adopt the same binding mode. Fragmenstein either takes the atomic coordinates of ligands from a experimental fragment screen and combines the atoms together to produce a novel merged virtual compound, or uses them to predict the bound complex for a provided molecule. The molecule is then energy minimised under strong constraints to obtain a structurally plausible conformer. The code is available at https://github.com/oxpig/Fragmenstein.

**Scientific contribution**

This work shows the importance of using the coordinates of known binders when predicting the conformation of derivative molecules through a retrospective analysis of the COVID Moonshot data. This method has had a prior real-world application in hit-to-lead screening, yielding a sub-micromolar merger from parent hits in a single round. It is therefore likely to further benefit future drug design campaigns and be integrated in future pipelines.

**Graphical Abstract:**

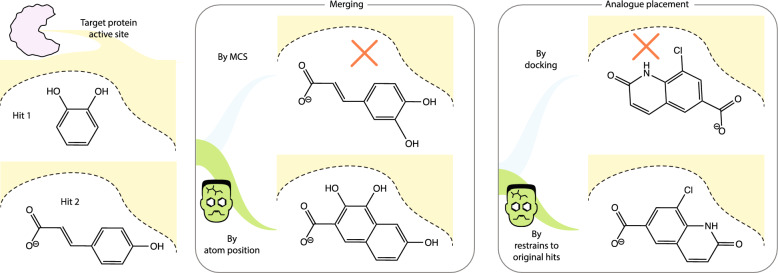

**Supplementary Information:**

The online version contains supplementary material available at 10.1186/s13321-025-00946-0.

## Introduction

### Fragment-based drug discovery is a standard methodology in drug discovery that leverages the similar binding mode between analogues

Fragment screening is an established approach in drug discovery to identify chemical moieties that will provide efficient binding either as starting points or as improvements to a lead [1–7]. It uses molecules under 250 Da, termed fragments, under the assumption that the information from multiple fragments is more constructive than the uneven information from a lesser number of standard small molecules with a higher molecular complexity as typically used in traditional high-throughput screening [4–7]. This is because of three principles. (i) Fragments are more likely to have a greater proportion interactions with the protein per atom than the per-atom proportion in standard (drug-like) small molecules, where several parts of the molecule may not interact with the protein at all [1, 6]. (ii) Fragments are likely to have a lower molecular complex than drug-like molecules and consequently a screen of a well-designed library will better cover chemical space [4, 5, 7]. (iii) Fragments are likely subjected to less strain than larger drug-like molecules, which often can explain sub-additivity in hit optimisation rounds [6, 8]. Based on these assumptions, it should be possible, as part of the fragment-based drug discovery (FBDD) design process, to take the protein–ligand interaction information from these smaller proximal molecules to design larger derivative molecules. This should result in the more efficient design of molecules which possess better binding affinity at a lower cost than lead optimization through structure–activity relationship (SAR) exploration of larger initial hits [6, 8].

Regardless of whether constructive structural information is available for initial fragment hits, by far the most common first-pass strategy is to enumerate derivative virtual compounds independently of structure, often through similarity or substructure searching, and afterwards employ docking as a conformational filter [9]. As discussed below, the shortcomings of this approach negatively affect successfulness of the searches.

### Unconstrained docking approaches as conformational filters do not fully leverage information from existing protein–ligand structures when predicting the conformation of derivative ligands

A common method to assess the binding of a candidate molecule is docking. Docking protocols consist of a search algorithm that performs thousands of heuristic iterations assessed by a score function to find the lowest energy predicted position, orientation, and conformation of the ligand in the context of the target protein [10]**.** Docking protocols find the energetic minimum according to the parameters of the force-field used to approximate the system, but may result in a local energy-minimum conformation that deviates from the one found in the experimental structure. This can occur for a variety of reasons ranging from insufficient or inaccessible sampling of either the ligand or protein conformations to inaccuracies of the physics in the empirical models. A common benchmark to assess the quality of a docking protocol is to “redock” the ligand from an X-ray crystal structure; namely removing the ligand and docking it and comparing the RMSD between the original and the docked ligand. With this approach, even the best algorithms reproduce roughly only half of all ligands docked to an RMSD of less than 2 Å [11]. An approach to improve this poor fidelity to the parent hits is by adding constraints to pharmacophores or to key atoms on the protein [12]. Another limitation stems from the fact most docking algorithms generate a set of small molecule conformers before docking which, especially for larger and more flexible small molecules, may all greatly diverge from the empirical crystallographic protein-bound conformer. Whereas it is straightforward to embed the conformer of a derivative ligand with the conformation of a parent FBDD hits that is its direct substructure, it is non-trivial when the substructure overlaps are imperfect and between multiple hits, as will be addressed below**.**

### Merging/linking approaches either disregard the position of hits or are unable to operate with overlapping hits

#### When ligands are designed starting from fragment hits (rather than docking a subset of virtual compounds in a dataset), the protein–ligand complex data available from initial fragment hit structures are often still not utilised until after initial enumeration

Three routes exist to elaborate one or more fragment hits: merging, where substructures of overlapping fragments are mixed, linking, where two non-overlapping fragments are joined, and growing where novel moieties are added to a starting fragment. These can be achieved in a variety of ways. Whereas, the latter adds new chemical matter, merging and linking approaches are mainly driven by pre-existing chemical matter, and are the focus of this work.

Approaches are usually synthon-based, where molecules are broken down into components and then new molecules are designed by mixing of components from multiple input ligands. Examples include BRICS decomposition [13] and AutoGrow4 [14]. Neither of these methods consider any 3D structural information from the protein or ligand in the initial enumeration step.

Some methods do consider some spatial information from the protein. DeLinker [15] is an example of a method which takes advantage of the 3D structural information of known ligands by identifying connection vectors between ligands and generating molecules that will fit into that 3D ligand space. However, it is still unaware of the protein environment around the ligands it is designing from. GANDI takes protein coordinates into consideration to filter out potential clashes [16], whilst designing linkers in a similar manner to DeLinker. DEVELOP takes this a step further by encoding both protein and ligand conformation into both connectivity (via a graph neural network) and coordinate information (through a voxel occupancy map) in its training to encode pharmacophoric features that can be used to predict new molecules for a protein target not in its training dataset [17]. STRIFE improves upon the predictions made by DEVELOP by also performing docking constrained to hotspot maps to better assess the products after a coarse-grain and a fine-grain step [18].

None of the methods discussed thus far consider the 3D conformation of overlapping hits. An algorithm that stands out in this respect is BREED [19], implemented within Maestro in the Schrödinger suite, this algorithm joins fragments hits by hybridizing upon spatially overlapping bonds, thus obeying the conformation of the hits. However, it’s a commercial product. In practice fragment merging is commonly done by eye [20].

#### Fragmenstein generates energetically feasible protein-bound conformers that obey one or more parent hits

To address the above problems, we developed Fragmenstein. The governing idea behind Fragmenstein is striving for fidelity to the position of the inspiring hits based upon the assumption that the derivative ligands bind in a very similar way. The crucial difference is that the conformers are generated by combining the coordinates of the atoms of the parent hits for both de novo generation (merging and linking), and for docking-like approach (placement), and subsequently minimised in place. To achieve this several tactics are employed to overcome certain issues, such as mapping partial overlaps to multiple parent molecules, merging rings and correcting impossible topologies. Fragmenstein can be used as a command line tool to automatedly place or merge/link combinatorially a list of ligands or be called within Python to merge or place ligands with custom mapping for more complex operations, such as flipping rings, or auxiliary operations, such as warhead conversion or further data analysis.

## Implementation

### Availability and requirements

The Fragmenstein codebase is a modular Python package that is dependent on RDKit [21] for molecular manipulation, optionally PyRosetta [22] for energy minimisation and some additional open-source purpose-written packages described in the GitHub repository. Its usage does not require external system calls, including the ligand parameterisation for Rosetta, which was rewritten to be both open source and usable within Python 3.6 + . Thanks to the limited number of external dependencies, it can be easily deployed in both Linux and MacOS architectures. It is designed to be used either in an interactive/library mode, including custom displays in Jupyter notebooks, or as a command line tool.

### Fragmenstein is open source

The open-source codebase (MIT-licence) for Fragmenstein can be found at https://github.com/oxpig/Fragmenstein.

Code and data for benchmarks (vide infra) available at https://github.com/matteoferla/Fragmenstein-manuscript-data.

Full documentation can be found at https://fragmenstein.readthedocs.io/.

### Fragmenstein merges ligands or places candidate ligands by using the coordinates of the atoms of the hits

**Fig. 1:**
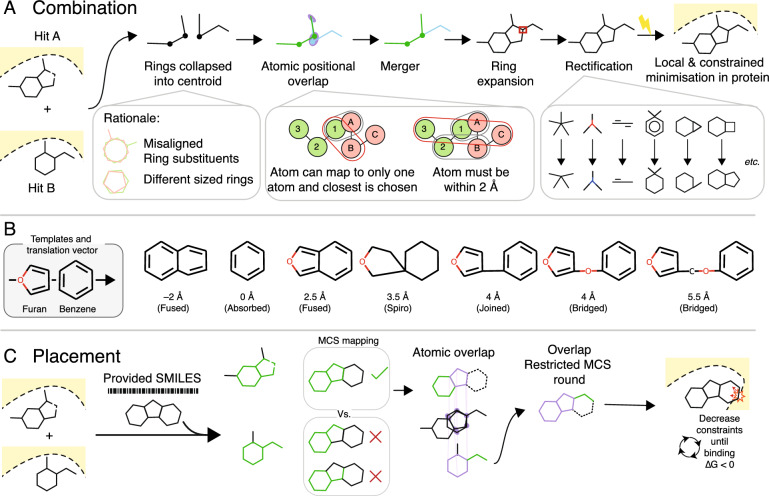
Combination and placement operations and their rules within Fragmenstein.** A** Steps in a combination operation. For combinations, the positional overlap is calculated with any ring collapsed. This is done to prevent overlap issues (first inset, detail in SI Fig. 1). Both rules share the atomic positional overlap mapping (middle inset, further detail in Supplementary Fig. 1). After which, the merger is rectified based on certain rules listed in its GitHub repository.** B** The effect of adherence to atomic positions can be seen in a test where a furan and a benzene with centres of mass at different distances yield different molecules ranging from a single ring to two linked rings (linking first atom set to oxygen).** C** Steps in a placement operation. The provided compound is mapped to each hit with a multistep MCS scheme (Supplementary Fig. 2), the mapping with the larger coverage is chosen and the other hits are mapped via a MCS restricted by their atomic overlap with the primary hit. For both combination and positioning, after the ‘stitched-together’ conformer is created, it is energy minimised locally, with strong constraints and with a topology parameterised from an ideal conformer

Fragmenstein at its core has two routes (Fig. 1): fragment hit merging/linking, herein termed as combination, and derivative placement, both constrained by the fragment hits that inspired them. Both these operations require two phases: (i) the creation of a potentially distorted molecule whose atoms overlap the parent hits and (ii) the energy minimisation of the molecule under strong constraints. Phase (i) differs between the two, bar for the determination of the pairwise maps of the overlapping atoms of the parent hits (outlined in Supplementary Fig. 1); this is a one-to-one mapping within a threshold (default: 2 Å).

Merging/Linking can operate on one or more parent hits. In the case of more than two, a pairwise operation is done starting with the first parent hit, followed by the next unless too distant: in such case it is re-assessed last (default cutoff: 5 Å). The first step in merging/linking (enabled by default) is replacing atoms in each ring with a single placeholder at the centroid with atoms and bonding recorded within. The atoms in the second ring-collapsed molecule that are absent in the first are added to the first ring-collapsed compound and bonded according to the original bond order, if possible. When the ‘stitched-together’ molecule is disconnected, the two closest atoms that can be substituted are identified and linked with an alkyl chain (length: off-by-one number of atoms = distance/1.22 Å), with the first atom being a heteroatom (default: nitrogen). After all the parent hits are merged/linked, the ring placeholders are expanded, whereby the original atoms and bonds are returned and any new bonds added if allowed by valency. This molecule is corrected (‘rectified’) to be both RDKit-valid and more chemically feasible. Some corrections are severe: geminal substituted arenes are dearomatized, aliphatic atoms with valence issues are either protonated or shifted by a group, while ‘Texas carbons’ (pentavalent carbons) lose a bond.

For placement, the graph of the desired molecule is mapped to the parent hits in an iterative manner (Supplementary Fig. 2), wherein a restrictive maximum common substructure (MCS) search is performed against all hits, the largest is used as the starting core followed by further laxer MCS searches constrained by the already mapped atoms (or their overlapping equivalents in other hits) with the possibility of excluding up to N atoms (default 3) if these prohibit the englargement of the mapping. In the case of novel atoms with no inferred equivalent, their coordinates are taken from a generated conformer superposed to the three closest atoms.

The minimisation phase operates in multiple steps for both routes. The first step (enabled by default) is a minimisation in RDKit with a frozen cutout neighbourhood where the atoms are heavily restrained if exocyclic, moderately restrained if ring atoms, not restrained if amides/ureas/esters or novel atoms (linking or unmapped), and with a restraint against E/cis conformations if exocyclic secondary amides. This compound is either further minimised in the protein with PyRosetta (default), OpenMM or none (halving the computation time relative to PyRosetta). For the PyRosetta minimisation using the ref2015 scorefunction [23], multiple cycles are performed in which the ligand is minimised under strong constraints like those in the RDKit minimisation and the neighbour around the ligand (centroid to centroid = ligand length + 3 Å) is allowed to move. After each cycle the weights are halved until the predicted energy of binding is negative (a single snapshot difference of bound minus unbound states).

The two routes (Fig. 1) can be combined into a single continuous workflow. First, fragments are combined (merged/linked) with Fragmenstein, then purchasable analogues are found via a third-party server (NextMove SmallWorld—sw.docking.org) [24]. These candidate ligands are placed into the protein structure with Fragmenstein, and lastly are ranked[25] by a multiterm score intended for customisation (default weights penalise loss of interactions, novel atoms, poor ∆G_binding_, number of rotatable bonds, and favour number of conserved atoms and interactions). Further details are available in the Supplementary Information and in the documentation. Additionally, several utilities are present, such as a wrapper for PLIP [25], functions for PDB preparation, covalent warhead handling, and visualisation.

## Methods

### Combinations on test datasets were conducted to assess success rate and availability from make-on-demand space

The hits from the XChem targets SARS-COV-2 MPro (cysteine protease) [26] and Mac1 domain of SARS-COV-2 NSP3 (macrodomain ADP-ribosylhydrolase) [27], were downloaded from Fragalysis (https://fragalysis.diamond.ac.uk/) [28] and filtered for inclusion in the DSi-Poised library [29]. The templates used were PDB:6LU7 for MPro and PDB:6WOJ for Mac1, these were energy minimised with PyRosetta with the FastRelax mover constrained by its density-map [22]. Their hits were merged/linked with the aim of quantifying the failure rate and the synthetic accessibility. Additionally, to explore the thermodynamic cost of fidelity to the reference ligands, as predicted by the Rosetta ref2015 scorefunction, alternative approaches were adopted, namely merging solely by maximum common substructure and merging by BRICS decomposition [30]. These were placed with the PyRosetta framework of Fragmenstein (Igor). BREED [19] was also run with 1.5 Å cut-off and with the “untangle” setting disabled to increase number of virtual compounds generated even if overly connected, but the limited results precluded its benchmarking. Interactions were determined with PLIP [31]. Interactive pages of results were created in Michelanglo [32].

### MPro was used to assess the accuracy of placements of derivative ligands

The information of which fragment hits were parents for which crystallised derivative ligands was taken from the Moonshot GitHub repository [26], but was reduced to contain only the relevant parent hits for each submitted ligand as these are presented together for each submission set. Namely, the relevant hits were manually picked based on the binding of the hits and the 2D representation of the derivative to not bias the selection (*cf,* code in repository). The common protein template used was PDB:6LU7 (substrate-bound form), which was minimised as describe above. Fragmenstein was run with the tweak that the PyRosetta Pose instance was modified to have catalytic His41 protonated on Nδ (HID) and Cys145 deprotonated for non-covalently bound ligands, while for ligands with electrophilic warheads His41 protonated on Nε (HIE) and Cys145 crosslinked with the ligand. Note that the latter functionality is automatic in Fragmenstein if the SMILES to be placed has a dummy/wild-card atom (* in SMILES, R in SDF) or the warhead conversion code within Fragmenstein is called.

RDock was used as a benchmark for pharmacophore-constrained docking [33]. executed on the same Mpro merges that were placed with Fragmenstein. For each ligand, the protein cavity was defined using the RbtLigandSiteMapper on the largest parent fragment hit with a radius of 8 Å and the following parameters: SMALL_SPHERE 1.0; MIN_VOLUME 100; MAX_CAVITIES 1; VOL_INCR 0.0; and GRIDSTEP 0.5.

One hundred poses per ligands were docked using the default “dock.prm” protocol. The top poses were selected based on the rDock score and the best RMSDs.

For the case of constrained docking, we computed the pharmacophores of the hits and set them as optional restraints with weight 1. The percent of constraints that should be satisfied was set to 80% based on a preliminary calibration test to achieve the lowest RMSD from the crystallographic pose. In a real-world scenario this calibration strategy is not possible since the crystallographic poses are not available, consequently, the results presented here are likely an overestimation of the actual performance.

### Two examples were retrospectively analysed, specifically addressing covalently bound ligands and user-provided mapping

First, to demonstrate the need for the thermodynamic corrections (minimisation) in the final step of Fragmenstein, the placement of a pair of derivative ligands binding NUDT7 from [34] (deposition group G_1002045) were investigated. NU181 (PDB:5QH1, chemical component: H5G, Enamine: Z1632454068) and PCM-0102716 (PDB: 5QH9, chemical component: GZY, Enamine: Z254513422) were the parent hits for NU443 (PDB: 5QHH, chemical component: H5D, S enantiomer) and NU442 (PDB:5QHG, chemical component: H17, R enantiomer), which were modelled with the chloroacetamide reacted with Cys73.

Second, to demonstrate the use of user correction, the placement of the derivative ligand binding the tubulin interface from [35] (deposition groups G_1002173 and G_1002214) was investigated. F04 (PDB: 5S4O, chemical component: O0J, Enamine: Z48847594) and F36 (PDB: 5S5K, chemical component: S6V, Enamine: Z2472938267) were the parent hits for todalam-4 (PDB: 5SB3, chemical component: 47F, Enamine: Z48853939). The modelling was done with a custom map in order to flip the N and S atoms in the aminothiazole (an equally plausible orientation given the electron density and required for the elaboration).

## Results

### A retrospective placement of 100 ligands by Fragmenstein based on their parents has much strong agreement with the crystal structures than that obtained by docking with pharmacophoric constraints

**Fig. 2:**
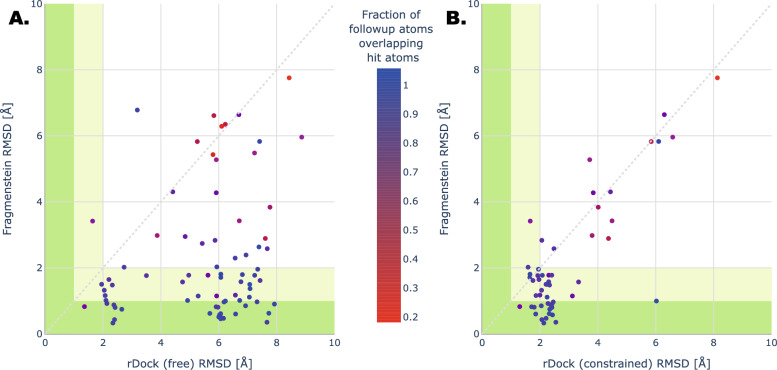
Accuracy of placement of Covid19 MPro1 Moonshot ligands. Derivative ligands in the Covid19 MPro1 Moonshot project which had a stated parent (manually adjusted) were placed with Fragmenstein and docked with rDock either freely (panel **A**) or with pharmacophore constraints (panel **B**). The initial dataset contained 87 fragment-derived ligands, but 8 were excluded due to lack of overlap with the parent hits and 20 were excluded in panel** B** due to failure to obtain a valid docking score for all replicates. Green area < 1 Å RMSD against crystal structure, pale green < 2 Å RMSD. Individual models can be investigated at https://michelanglo.sgc.ox.ac.uk/r/fragmenstein-moonshot

A key underlying hypothesis is the derivative ligands bind in a very similar way to their parent fragment hits. Fragmenstein merges fragments by first combining the positioned atoms of the parent fragments first and then locally minimising under strong constraints, without relying on previously generated conformers. We hypothesise that constrained minimisation, as occurs in Fragmenstein, is more effective than unconstrained and pharmacophore-constrained docking at predicting the pose of elaborations based on parent fragments. To test this, a dataset of matched parents–elaborations was constructed from the Covid Moonshot project data, since this contains a large panel of hit-inspired derivative ligands [26]. The Covid Moonshot project was a collaborative SAR-COV-2 protease fragment-based drug discovery project that relied on an automated crystal soaking pipeline and on user submitted ideas of derivative ligands. These submissions were guided by user’s choice and as a result represent a spectrum of diverse approaches. The submissions were filtered for ligands that were crystalised and that had two or more stated parents, yielding a total of 87 ligands, 65 of these were cases designed so one fragment contributed a single substituent, while the remainder were more balanced designs. The atomic positions of the conformer from the crystal structure were compared to those of a conformer placed by Fragmenstein constrained against the stated inspiring hits and to those of conformers docked with and without restraints (Fig. 2, interactive at https://michelanglo.sgc.ox.ac.uk/r/fragmenstein-moonshot).

The importance of exploiting the structural information of the parent hits is illustrated by the fact that out of the 87 elaborations, 79% (69/87) have some overlap with the parent hits, 69% (60/87) were found to preserve the pose of their parent fragments (combined RMSD < 2 Å), and 40% (35/87) persevered it well (RMSD < 1 Å).

Fragmenstein was able to propose high-quality poses (RMSD < 1 Å) for 28% (24/87) of the evaluated ligands and acceptable poses (RMSD < 2 Å) for 56% (49/87) of them. Docking (via rDock) was able to obtain only 3 poses with an RMSD under 2 Å (1.95, 1.35, 1.63 Å) (Fig. 2A).

In the cases were Fragmenstein failed, the ligands bound in the same pocket as the hits but the Fragmenstein models had an RMSD > 5 Å were × 2581 × 10,236 × 2764 × 10,900 × 2779 × 1386 × 3305 × 1384 × 10,606× 10,723 × 10,049 × 3366, for these either the crystallised ligand disobeyed the hits or Fragmenstein incorrectly mapped the derivative to the hits due the convoluted overlay. With the caveat that predicted Gibbs free energy of binding is not a strong metric (vide infra), this correlated with the shape overlap of the hits and the crystal pose (− 0.35 ± 0.16), indicating that worse scoring mergers tend to preserve less the binding mode.

In order to determine if Fragmenstein was able to better exploit the structural information of the fragment hits than other approaches, we next compared Fragmenstein with the constrained version of rDock using pharmacophoric constraints derived from the parent hits. Figure 2B shows that, while including constraints improves the docking performance, Fragmenstein still outperforms rDock, which was able to produce poses within 2 Å RMSD for 20% (14/67, vs. 47/67) of the ligands. A factor involved is that Fragmenstein generates the conformer based on the hits, while docking frequently choses a conformer among a set of generated conformers. Specifically, for this dataset, the most similar generated conformers out of 10, 100 and 1,000 (ETKDG in RDKit) to the crystallographic pose deviated by 0.9 Å, 0.7 Å and 0.6 Å on average. The inability to sample a conformer that perfectly matches the crystallographic one underlies the choice in Fragmenstein to start from a ‘stitched-together’ conformer. This together with the hit-derived strong constraints during minimisation allows the placed molecule to be highly faithful to the parent hits.

### On two datasets, Fragmenstein proposes 31 and 24 easily accessible derivative virtual compounds (in catalogue or with catalogue-analogues with graph edit distance of 2 or 1) from the merging/linking of 34 and 44 parent hits

To assess the overall quality of combinations (mergers/linkages) from Fragmenstein, *i.e.* determining the methodological failure rate and synthetic accessibility, two targets, MPro (a cysteine protease from SARS-COV-2) and Mac1 (a nucleosyl-peptide hydrolase from SARS-COV-2) from previous fragment screens were chosen and the initial hits that originated from a library designed to facilitate synthetic derivatives (DSi-Poised) were combined (merged/linked) and scored (Table 1, interactive at https://michelanglo.sgc.ox.ac.uk/r/fragmenstein-mpro-DSiP). Fragmenstein is able to perform mergers of more than two ligands, however, in a benchmarking combinatorial experiment, it is not advisable due to combinatorial explosion, molecular weight increase, and understandable lower number of analogues, consequently, only two way merging/linking was performed. Excluding the combinations that were over 5 Å apart for their closest atoms, the failure rate was 1.4% due to ligands whose chemistry could not be rectified correctly, while 56% of combinations were predicted, by the Rosetta scorefunction, to be energetically favourable (∆G_bind_ < 0) without excessive deviation from the positions of the parent hits (RMSD < 1.). Of the 420 acceptable combinations, 7 were purchasable, while 64 could potentially be made with 2 or fewer reactions according to predictions from PostEra Manifold [36]. Therefore, Fragmenstein suggests synthetically accessible virtual compounds that are predicted to follow the binding conformation of the parent fragment hits, which is an underpinning assumption in fragment-based drug discovery (*cf.* Figure 1).

**Table 1:** Assessment of the quality of mergers generated with Fragmenstein. Combinations (mergers/Linkages) were computed for DSiPoised subset of hits for the targets and classified by outcome and then the acceptable molecules were further assessed for synthentic accessibility

	MPro	Mac1
Number of hits used	34	44
Number of acceptable^a^ mergers	157	263
Number of failed mergers due to equal size to one hit	13	34
Number of failed mergers due to > 5 Å minimum distance between hits	918	1438
Number of failed mergers due to strain (∆G_bind_ > 0 kcal/mol or > 1 Å RMSD)	33	149
Number of failed mergers due to technical issues	1	8
median mol. wt of acceptable subset	356.1	305.0
median QED ^b^ of acceptable subset	0.79	0.66
Number of of acceptable molecules with SA ^c^ < 0	54	27
Number of of acceptable molecules with SA ≤ 0.4	71	40
Number of acceptable molecules that are purchasable^d^	5	2
Number of acceptable molecules with purchasable analogues in Enamine Real differing by 2 or 1 atoms	26	22
Number of acceptable molecules accessible via a one-step synthesis^e^	28	10
Number of acceptable molecules accessible via a two-step synthesis	16	10

a) The acceptability criteria were both hits were used, RMSD < 1 Å, ∆G_bind_ > 0 kcal/mol, and number of heavy atoms greater than that of the largest. hit,

b) QED: Quantitative Estimate of Druglikeness, calculated by RDKit

C) SA: FastSAScore calculated by Postera Manifold

D) Purchasable: molecule available from the vendors Enamine (BB, MADE and REAL), Sigma, Mcule, EMolecules, Molport, WuXi (BB and GalaXi)

E) 1-step / 2-step: Molecule unavailable but synthesisable in a one or two reactions as predicted by by Postera Manifold retrosynthesis. The combinations can be inspected at https://michelanglo.sgc.ox.ac.uk/r/fragmenstein-mpro-DSiP

### The strict obedience to atomic positions by Fragmenstein is a strong filter whose effects may be misled by potentials and are unmasked when counting number of interactions

As described above, a key point of Fragmenstein is obedience to parent hits. To emphasise the importance of fidelity to conformation of the parent hits, the initial hits of Mac1 were merged/linked pairwise ignoring positional information in three different approaches. In one the parent hits were merged by maximum common substructure (MCS), in another by BRICS decomposition, and in a third with Fragmenstein but constrained to a single hit.

With the caveat that larger numbers of valid virtual compounds does not mean potential ligands with higher affinity, the former was used as a test metric for illustrative purposes. The minimisation of MCS and BRICS mergers in place via constraints to both the parent hits (regular Fragmenstein) did not yield any acceptable poses, whereas the minimisation in place against only the larger hit resulted in a jump to 23% for MCS and 34% for BRICS (Figure S3). When Fragmenstein mergers were constrained to a single hit, the acceptance rate increased from 11 to 14%, because several mergers that were irreconcilably strained when constrained against two hits were more relaxed when constrained against a single hit and not obliged to respect the position of the second hit.

The number of interactions formed as determined via PLIP reveals a median 0.25 interactions per heavy-atom count (HAC) for the acceptable two-hit–constrained Fragmenstein mergers and a lower 0.21 interactions/HAC for single-hit–constrained Fragmenstein mergers.

This is because without the positional constraints the force-field dominates the placement by pushing towards a distant energy minimum. Fragmenstein utilises molecular mechanics but does not find the energy minimum within a box, and instead finds a low energy state around the initial hit. As a consequence, the calculated free energy of binding are sensitive to the number of constraints applied and are not an overly meaningful metric. A common metric that disentangles binding energy from atom count is ligand efficiency (LE, nominally in –kcal/mol/HAC, but effectively unitless [37]). Unsurprisingly the median LE improves from 0.20 for the two-hit–constrained mergers to 0.23 for the single-hit–constrained mergers, despite the latter forming less meaningful interactions by not obeying the conformation of the second hit.

The pure-MCS mergers constrained to the largest hit had both fewer interactions and worse free energy of binding (median ligand efficiency of 0.14) due to the more compact nature, making the mergers more likely to fall off an energy cliff. This contrasts with BRICS decomposition where the substructures of the parent hits are joined at the broken bonds therefore respecting the axis of the parent hits, even if they may not have been spatially overlapping. In the BRICS approach, the constraints were to a substructure of single hit, so the ligand efficiency is better than Fragmenstein (0.25), whereas the median number of interactions was actually lower (0.17 interactions/HAC).

**Case examples**

### Fragmenstein can work with covalently bound ligands

To work with covalently bound compounds, Fragmenstein treats the attachment atom (stored as a dummy atom) and defined atoms from the warhead differently, primarily by protecting these during merging. To test the impact of having a covalent attachment, the placement of a published ligand [34] with two stereoisomers was replicated. In this study, only one enantiomer reacted with the thiol of the catalytic cysteine in the protein (NUDT7).

**Fig. 3:**
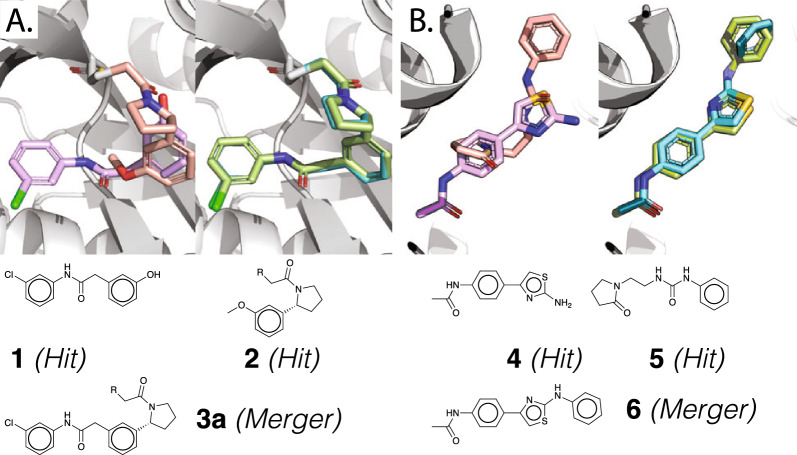
Retrospective Comparison of crystallised and placed derivative ligand from NUDT7 study **A** and tubulin **B** study, illustrating a merger with hits that do not overlap cleanly and a merger requiring a user-defined mapping respectively. In the NUDT7 study, the two fragment hits PCM0102716 (**1**, coral, LHS) and NU181 (**2**, in lavender, LHS) were merged by Resnick et al*.* yielding the merger NU443 (**3**) [34]. The crystal structure of **3a** (turquoise, RHS) overlayed with the placement predicted by Fragmenstein (green, RHS). **2** and **3a** are covalently bound with Cys73 via an warhead. Internally outside of the PyRosetta operations, covalent attachment atoms are stored as dummy/R/* atoms, shown in white.In the tubulin study (pane B), F04 (**4**, lavender) and F36 (**5**, coral) inspired Todalam-4 (**6**, sky-blue: crystal, green: predicted). The aminothiazole ring is flipped between **5** and **6** by design. A constructive observation of this derivative is that the N-benzyl is rotated in the crystal relative to **4** possibly to attain a T-shaped pi bond, a dipole-momentum–driven configuration, which is not modelled in classical mechanics forcefields such as that employed by Rosetta

This ligand is merger of two hits (**1**, NU181 and **2**, PCM0102716) which were used for placement with Fragmenstein. The RMSD between the placed model and the crystal structure of the merger is 0.28 Å, while the aggregated RMSD values of the model and the structure against the parent hits are 0.65 and 0.61 Å, indicating that the slight conformational change resulting from the constrained minimisation is also seen in the crystal structure. This placement (Fig. 3A) operation also showcases a feature of Fragmenstein borne out of having to operate on multiple hits. Namely, that some superposed substituents in the hits may act as red herrings and are ignored, in this example the hydroxyl of one hit (**1**) is automatically ignored from the mapping as it would otherwise impede the mapping of the second hit (**2**) which has a group occupying the same space. In this fragment screens, as is common, a racemic mix first soaked in the crystal (NU308) and was subsequently chirally separated into two stereoisomers (**3a**, NU443 and **3b**, NU442). Whereas one stereoisomer (**3a**) was found covalently bound, the other (**3b**) was found not reacted. Placing with Fragmenstein the latter stereoisomer as a covalently bound compound compound (Supplementary Fig. 4), yielded a pose with a 10% worse binding ∆G (predicted by Rosetta ref2015 scorefunction without constraint weights) than the former and with a 0.9 Å shift in the sulfur atom of the connected cysteine relative to the position in the parent hit, indicating that the covalent bond is expected to be strained as is confirmed in the crystal structure wherein the presumably worse reaction barrier was not overcome.

### In Fragmenstein, it is possible to enforce derivative atoms to map to specific atoms from the hit atoms in order to get the intended placement

An example of this is a parent hit with a ring in a flipped conformation. Crystallographic structures generally consist of a single conformer bound in a set orientation as suggested by the electron density map. In some cases, for example with the terminal amides of glutamine or asparagine or the ring in a histidine, the specific density alone cannot reveal which way these sidechains are oriented. This can apply to ligands [38].

An example of this is seen with tubulin inhibitor Todalam-4 (**6**) [35]. This ligand is the merger of two fragment hits (**4**, F04 and **5**, F36). One possesses an aminothiazole ring placed in one orientation in the crystal structure, yet for the merger to be accurate, the flipped orientation is required. Fragmenstein will determine the ring to be not productive and ignore it, however the user may want to manually enforce the ring mapping. When applied to this test case, when passed a map to override certain atoms Fragmenstein correctly predicts the intended placement (Fig. 3B). This ability to fine tune the behaviour of Fragmenstein allows it to be highly versatile and adaptable.

## Discussion

### Elaborations empirically follow their parent hits, so designs ought to do the same

The core principle of Fragmenstein is to create a conformer of a molecule, via its two routes (combinations or placements) by combining the atomic positions of the parent hits, with the aim of being as faithful as possible to these without being energetically unfeasible.

Docking is often employed to shortlist compounds, however, when used without tailored constraints, it has the problem that the outputted conformer may not reflect the binding of the fragment hits that inspired them, even though fragment hits with a common substructure are most often found positioned in a very similar manner [39]. Were a docked derivative candidate to interact differently than its parents, the validity of its score would be rightfully put to question by an experimentalist. Several decomposition studies address the SAR additivity/superadditivity of certain functional groups [8, 40–43] and how the binding mode is maintained crystallographically. Here, the inverse direction is taken and is found also to be consistent; in Fig. 2 it was shown that in the Covid-Moonshot dataset of the crystallised derivative compounds that bound similarly to their parent 69% are placed by Fragmenstein with an RMSD under 2 Å compared to 20% by pharmacophore-constrained docking. Confirming the importance of obeying the position of the atoms in the parent hits.

Fragmenstein has a very high success rate in combining (merging/linking) parent hits and yields several virtual compounds in make-on-demand space (Table 1). Fragmenstein aims to preserve the interactions of the parent hits unlike other methods. Nevertheless, in assessing the elaborations, one may be misled by the metrics used. Predicted Gibbs free energy of binding can be misleading, especially when constraints are involved: reducing the number of constraints improves this metric, whereas there are fewer interactions.

In the test cases used, namely two hydrolases, one acting on a peptide and the other on a nucleotide modification, the pocket is restrictive. When the region of interest is large, as is often the case for protein–protein interfaces, in an unfocused scenario there will be an overwhelming number of acceptable purchasable analogues, which will require significant shortlisting.

### A simple energy score for exploration is unsuitable for shortlisting virtual compounds for purchase or synthesis

Ranking virtual compounds via a predicted energy metric is less than ideal in general: a principle that also applies to Fragmenstein. This is in part since predicted energy, even with more advanced methods, cannot perfectly predict binding affinity [44]. With Fragmenstein in particular, the energy estimate is not of a global energy minimum, but a minimum highly constrained to the RMSD between the placed coordinates and parent hits: the RMSD should therefore be considered alongside the predicted potential.

Even if the predicted binding energy were perfectly accurate, this would not the sole factor to consider. In a pipeline, where fragment hits are combinatorially combined, analogues identified by catalogue, and then placed, the next challenge becomes choosing which compounds to purchase, a problem shared with other methodologies. Three operations are commonly performed: filtering, sorting, and clustering. One possible filter is vendor driven, namely the removal of compounds above a given price point or with unworkable delivery times. Another possible filter is the wholesale removal of compounds with substructures that may cause assay interference, such as fluorescence or PAINS, or may be toxic (*e.g.* Ghose or REOS filters), or may not be drug-like (*e.g.* Lipinski rules) [45, 46]. Whereas sorting by predicted energy or similar score is the simplest approach, it is less suitable in the real world than a blend of different metrics in addition to score or number of interactions. One factor is risk, whereas a conservative elaboration may be more likely to bind, more information may be gained from a riskier derivative compound. A variety of other factors could be considered such as ligand efficiency, molecular weight, number of hydrogen bond donors, TSPA, logP, and a penalty for rotatable bonds, the latter on account of entropic loss from the decrease in degrees of rotational freedom upon binding. One further step, especially useful for hit discovery, is clustering by the interactions formed. A major criterion used in shortlisting is the relevance of given interactions in respect to the native biochemical mechanism that is aimed to be disrupted, for example substrate-binding in the case of enzymes. These various steps together better reflect a drug discovery campaign as they allow a set of virtual compounds with desired properties and diverse binding modalities to be shortlisted as opposed to simply by predicted energy.

### Fragmenstein can be paired with catalogue searches and decomposition

In an applied scenario, certain hypotheses/series can be problematic to explore due to the non-uniform distribution of fragment-hits or limited sociability of certain fragments: these would need addressing by complementary methods to merging/close linking, such as scaffold hopping, fragment growing, catalogue enumeration of superstructures to join two distant moieties and so forth. The linking approach is intentionally basic as Fragmenstein is not intended for Protac design (*i.e.* two distinct moieties tethered by a long flexible linker) or to add novel chemical substructures between two hits. These use cases are addressed by other tools [15, 17, 47, 48]. A recent published approach, for example for fragment joining enumerate all purchasable compounds that contain substructure of pairs of hits and places these with Fragmenstein [47].

An example of a case that Fragmenstein, or merging in general, is unable to tackle well is merging two perpendicular arenes overlapping by one atom: the merger is a spirocyclic compound, which may be strained, synthetically inaccessible, and majorly unable to bind due to the lack of aromatic properties, such as partial charge distribution and polarizability. For such cases, growing is a better approach.

For close compounds, the torsion of the link may be highly constrained by the substructures from the parent hits, which is exactly the sort of problem Fragmenstein can address as demonstrated in its role in aiding the identification of a IC_50_ 430 nM inhibitor against SARS-COV-2 Mac1 [27, 49], specifically the top three mergers/linkers between fragment hits in two critical pockets (adenine pocket and ribose/oxyanion pocket) were used in an analogue search (all mergers: https://michelanglo.sgc.ox.ac.uk/r/fragmenstein_nsp3).

Even though the compounds generated by combination are chemical correct, a limitation of this is that the compounds created may not be in make-on-demand space or may not be synthetically accessible. In the provided demonstration notebook the SmallWorld server is queried to find purchasable analogues from Enamine REAL (an analogues-by-catalogue approach) [24], which can be placed by Fragmenstein. A similar approach was used in the SARS-COV-2 Mac1 study[27] (using Arthor, https://arthor.docking.org/ [24]). Chemical make-on-demand space despite its vastness is often limiting. In fact, it should be noted that the outcome of the search may not be always fruitful. For example, a merger of two perfectly placed parents may yield a compound that is far removed from make-on-demand space (e.g. Supplementary Fig. 5, a clear planar merger distant from make-on-demand space), thus forcing the user to consider other mergers or linkers as a starting point for exploration. Predictably, the more the lead-like candidates grow, the more isolated they may be in easily synthesisable chemical space.

A fruitful synergism to optimise compounds is combing BRICS decomposition and Fragmenstein, which in effect removes substructures from the initial hits which are not forming good interactions or hamper synthetic accessibility.

Beyond drug discovery, Fragmenstein has found uses in biochemistry settings by virtue of allowing the change of a crystallographically amenable analogue for the native substrate, *e.g.* the non-hydrolysable guanosine imidotriphosphate (GNP) for guanosine triphosphate (GTP) [50].

## Conclusions

Fragmenstein is first and foremost a tool that strictly obeys the parent hits both as a generative model and as a docking alternative. This provides a way for a human user to drive their computational experiment to meet their hypothesis by controlling and appraising the prediction: in the end, the decision of which compounds to purchase is very rarely left to a blind algorithm and instead is put in the hands of an experienced chemist.

## Supplementary Information

Supplementary Material 1.

Supplementary Material 2.

## Data Availability

The code is available from https://github.com/oxpig/Fragmenstein, data is available from https://github.com/matteoferla/Fragmenstein-manuscript-data, and documentation is available at https://fragmenstein.readthedocs.io.
