# Supplementary material for: Fragmenstein: predicting protein–ligand structures of compounds derived from known crystallographic fragment hits using a strict conserved-binding–based methodology
Source: J Cheminform. 2025 Jan 13;17:4. doi: 10.1186/s13321-025-00946-0 (PMC11731148; doi:10.1186/s13321-025-00946-0)
Supplement: Supplementary file 1 — Supplementary Material 1. [file 13321_2025_946_MOESM1_ESM.pdf]

DETAILED WORKING OF FRAGMENSTEIN

For full documentation see: https://fragmenstein.readthedocs.io/en/latest/

OVERVIEW

MAIN ROUTES OF FRAGMENSTEIN

1. **Combination**: This involves merging and linking fragment hits based on their atomic overlap, creating a novel compound.
2. **Placement:** A given compound is positioned based on the positions of the parent fragment hits.

FEATURES AND FUNCTIONALITY

Fragmenstein uses several purpose-built external modules and includes various classes to perform its tasks. It can be invoked from the command line and can use either PyRosetta (default, via the `Victor` class), OpenMM (`OpenVictor` class), or RDKit (`Wictor` class) for energy minimisation. There are also variants tailored for speed, such as the `Quicktor` class.

ATOMIC MAPPING

Generating a stitched-together conformer (via the `Monster` class) requires mapping the atoms of the parent hits to each other, whether a desired compound (`place` route) is supplied or not (`combine` route). The mapping is done by atomic position rather than bond structure, which allows for greater flexibility but necessitates several hardcoded conditional rules to determine overlapping atoms.

Key rules for atomic mapping include:

- Each atom in one conformer is mapped one-to-one based on positions within a 2Å threshold for both combination and placement.
- A single atom from one fragment hit can only map to a single atom on the other hit.
- Mappings are pairwise, even when more than two hits are present, meaning three-way mapping may not be transitive.

CLASS HIERARCHY

The RDKit operations with two or more parent hits is performed by `Monster`.

```
monster = Monster([hit1, hit2])
monster.combine()
monster.positioned_mol
```

This class can additionally minimise in RDKit within an optional frozen pocket as called by Victor.

The class `Victor` relies on `Monster` and `Igor` (or `Fritz`, in the case of `OpenVictor`) to combine/place the parent hits and to minimise within the protein respectively.

The class `Laboratory` calls Victor iteratively to either merge/link all hits in pairwise or higher combinations, or to place compounds based on a provided dataframe. The class `Walton` is intended solely for illustrative purposes, as it generates synthetic data.

COMBINATION PROCESS

Combining compounds with superposed rings of different sizes can result in unnecessary strain and suboptimal bonding. Therefore, Fragmenstein substitutes each ring with a placeholder pseudo-atom, ensuring that rings only map to rings, preventing problematic distortions. Covalent attachment atoms are also matched exclusively with other attachment atoms. The overlapping atoms are then merged, with careful handling of ring-demarcating atoms and the creation of new bonds as needed. If the resulting molecule is disconnected, fragments are joined via the closest atoms, with a penalty for warhead atoms or fully bonded atoms, and linker atoms are added if necessary.

Ring collapse

To prevent distorted ring mergers, the default operation collapses the rings into a placeholder atom and expands it after merging (disable via `Monster(...,collapse_rings=False)`). The order of the hits provided is important as it determines which atom is used when two parent atoms overlap. When the collapsed ring is expanded after a merger of two ring placeholders, the original bonds are restored whereas substituents from the lower priority parent hit are connected based on distance to the closest partially substituted atom from the ring. In the case of substituents not close to a partially substituted atom, they are attached to the closest atom regardless, thus subsequently triggering the need for correction. For example, a superposed pyridine (top priority) and toluene with the aza nitrogen and methyl-substituted carbon overlapping, the resulting compound would be a N-methyl-pyridinium or toluene depending on the setting of the rectification process.

LINKER

Fragmenstein can link compounds up to a given cutoff distance, which is 5 Å by default, but can be changed (`$FRAGMENSTEIN_CUTOFF`, `Monster(…, joining_cutoff)` or `Victor.monster_joining_cutoff`). Parent fragment hits that are disconnected will be connected. When the two hits are more than 2 Å apart a linear series of atoms is added. The identity of the first is nitrogen or user specified (`$FRAMENSTEIN_LINKER_ATOM` or `Monster.linker_atom`), while the remaining are carbons. These atoms are not constrained.

The atom to which the linker is attached is the closest partially substituted atom.

This is very basic and we recommend tools that perform catalogue searchers to link more distance compounds.

RECTIFIER CLASS

The resultant molecule is corrected by the `Rectifier` class to address issues such as atoms exceeding their supported valence. Novel small rings, bridged rings, and allenes are removed as they are likely unnecessary. Users may then test the placement of similar, more synthetically accessible compounds.

the rectifier class performs the following operations:

- Corrects protonation where appropriate, such as the incorrect number of hydrogens on nitrogen heteroatoms in arenes.
- Corrects valence of Texas carbon by removing bonds that are too long or cause additional issues.
- Corrects valence by shifting the element symbol to one with a higher valence. In the case of nitrogen with valence 4, and if enabled, these atoms may be protonated.
- Reduces rings in cases where arenes fail the above corrections.
- Breaks novel cyclopropane rings if possible.
- Expands novel cyclobutane rings fused to other ring systems by one atom, resulting in a fused 5-membered ring.
- Removes bonds forming novel bridged rings, as they are challenging to produce synthetically.
- Corrects other specific strained groups, such as allene.
- Corrects all radicals. This rules-based approach ensures the creation of chemically valid molecules without imbibing synthetic accessibility (i.e., sanitize in RDKit).

This class was made for Fragmenstein, but is in separate repository (`molecular-rectifier`), as it broadly applicable.

this is because it can be used independently of the Fragmenstein pipeline, for example correcting compounds from de novo denoising diffusion models that do not sanitise in RDKit.

Placement Process

When placing a follow-up compound, atoms are mapped to the inspiration hits using maximum common substructures (MCS), iteratively adjusting the stringency until preset rules are met.

This includes constraints on bond lengths and the exclusion of misleading atoms to achieve fuller mapping.

In the case of atoms that are not present in the hits, these are embedded based off an superposed conformer of the desired compound,

this circumevents the requirement for the compound to energetically valid when performing a partial embedding in RDKit.

Minimisation

The "stitched together" molecule, likely in an energetically poor conformation, is minimized by the `Igor` class when using `Victor`,

or by `Fritz` when using `OpenVictor`. The former takes ~60s per compound on a single core, and is much faster than the latter, which requires a GPU. `Wictor` only does the RDKit minimisation (frozen pocket) and takes ~20s per compound on a single core.

This can be preceded by optional RDKit minimisation within a frozen protein pocket to limit clashes.

This approach differs from enumerating many conformers, focusing instead on refining a single distorted conformer to match the hits.

The parameterisation of the molecules for use in PyRosetta is performed by the class `Params` from the module `rdkit-to-params`, which was written purposefully for Fragmenstein to overcome the portability issues with the Python 2.7 module that is normally distributed with Rosetta for such a task and allowing API usage.

Igor uses FastRelax (in cartesian mode) with a custom protocol, wherein the constraints are unaltered during the cycles:

```
['repeat %%nrepeats%%',
f'coord_cst_weight {weight}',
'scale:fa_rep 0.092',
'min 0.01',
'scale:fa_rep 0.323',
'min 0.01',
'scale:fa_rep 0.633',
'min 0.01',
'scale:fa_rep 1',

'min 0.00001',
'accept_to_best',
'endrepeat']
```

Under default setting if the minimisation failed (*i.e.* positive predicted ΔG_{bind}) the weight of the constraint is halved and the minimisation is reattempted. When the weight is .005, one last attempt is made. If the attribute, `quick_reanimation` is True, then only one trial is done with 5 cycles (default 15). Before and after this minimisation ('reanimation'), the neighbourhood with centroids with 4 Å + size of ligand are repacked.

The score is calculated with a ref2015cart scorefunction without weights for the complex subtracted by the ligand in isolation and the apo form of the protein.

PIPELINE

The class `Laboratory` automates the analysis of a set of fragment hits.

- Combinations. All permutations of merging/linking at a specified k-arity (default: pairwise) for the provided hits
- Placements. Places all compounds based on provided parent hits in a given table

Additionally, special operations are present, such as all the mergers/linkers of one set with a second set. The output of these is a Pandas dataframe for filtering and ranking. The class `Victor` can call the tool PLIP for a list of interactions with the protein residues. This is used in the output of the class Laboratory for scoring, wherein it can be used by the user for filtering (e.g. for kept interactions for example).

In fact, in addition to generating the list the class also clusters and assigns a weighted linear sum of scores (`ad hoc penalty`) based on properties set by the user. This is technically a penalty as negative is good, in order to be consistent with binding potential. This is done by the `score` method,

which can be called repetedly to fine tune the weights for each property for better ranking of the virtual compounds. The default values are:

- +1.0× ΔGbind (negative is good)
- +1.0× number of rotatable bonds (the entropic penalty of rigidification cannot be accounted for in a static snapshot model and is ~0.6 kcal/mol)
- +5.0× interaction uniqueness (probability scaled sum of interactions)
- +0.2× number of unconstrained atoms (to counter novelty winning)
- –0.05× number of constrained atoms (a counter balance to the above)
- –1.5× number of interactions
- +2.0× number of interactions lost relative to parent hits
- +5.0× number of PAINS matches
- +1.0× strain over number of heavy atoms

Additionally, Butina clustering by Tanimoto similarity is also performed.

Together, these allow the virtual compounds to be ranked to suit the needs of the user to best balance diversity, novelty and risk.
