# Supplementary material for: Fragmenstein: predicting protein–ligand structures of compounds derived from known crystallographic fragment hits using a strict conserved-binding–based methodology
Source: J Cheminform. 2025 Jan 13;17:4. doi: 10.1186/s13321-025-00946-0 (PMC11731148; doi:10.1186/s13321-025-00946-0)
Supplement: Supplementary file 2 — Supplementary Material 2. [file 13321_2025_946_MOESM2_ESM.pdf]

Supplementary Figures

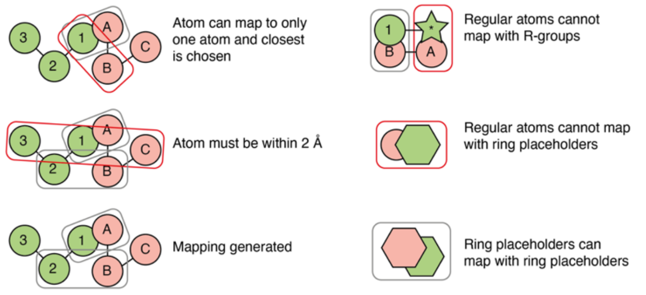

Supplementary Figure 1. Detailed rules employed in determined atomic overlap between two compounds. Atoms cannot map to multiple atoms and cannot map spanning a distance greater than 2 Å. R-groups (dummy atoms) marking covalent attachment points can only map to other R-group. Likewise, ring placeholders cannot map to regular atoms.

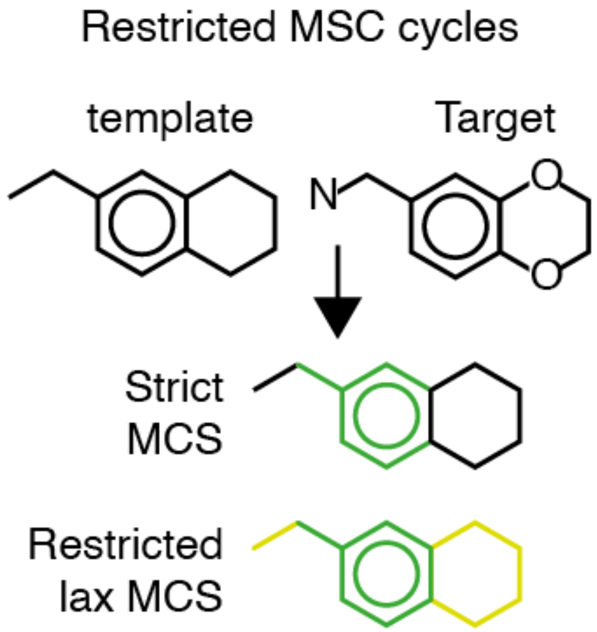

Supplementary Figure 2. Detailed mapping schema used in the placement operation. As the compound that is to be mapped onto a fragment hit may contain differences, primarily as expansions, relative to the reference compound, multiple round of maximum common substructure mapping are employed. First a strict mapping is performed, then a series of more lax mappings are performed constrained on the first mapping in order to cover the most atoms, while still maintaining the core details. When mapping a compound to multiple hits, the mappings covering the largest number of atoms is likewise used to constrain the mapping of the other hits.

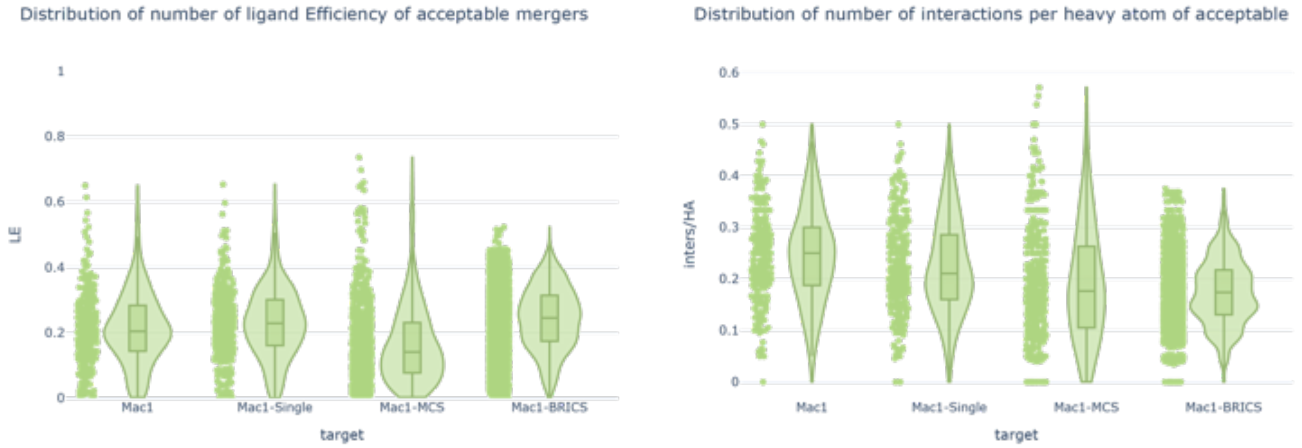

Supplementary figure 3. Distribution of ligand efficiency (left) and of number of interactions per heavy atom for the different merger performed on the Mac1 poised dataset, namely Fragmenstein, Fragmenstein modified to be constrained to a single hit, MCS merger (void of positional information) and BRICS decomposition and building. The median (centre line within each the box) for BRICS and for the single-hit–constrained Fragmenstein are larger than the two-hit–constrained Fragmenstein median in the ligand efficiency plot, but not in the interaction count plot because the minimisation for the regular Fragmenstein is more constrained thus affecting the calculated Gibbs free energy of binding.

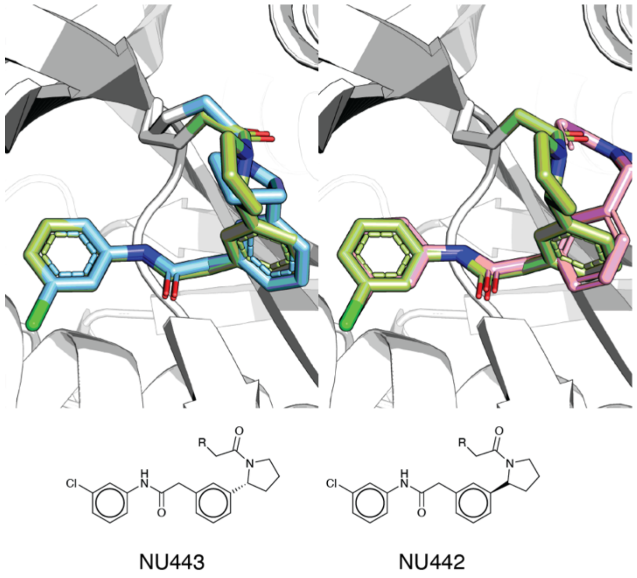

Supplementary Figure 4. Placement of NU442. Turquoise: hits, green: Fragmenstein-generated conformer (covalent), puce: crystallographic conformer (unreacted). The sulfur atom is shifted in the Fragmenstein conformer relative to the parent due to the strain imposed by the chirality of the pyrrolidine substituent.

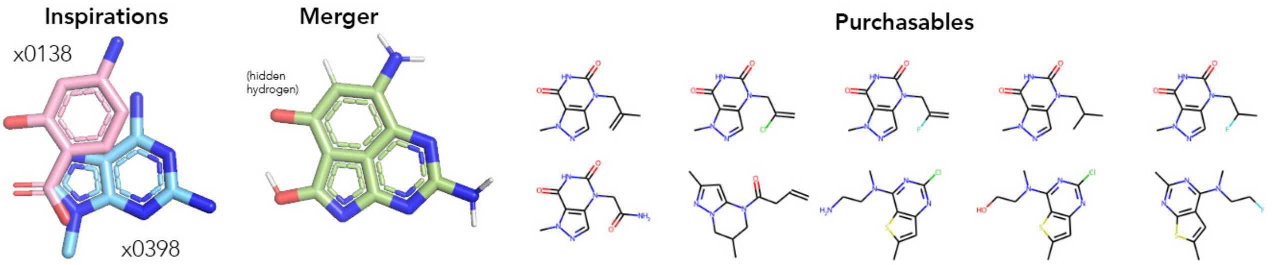

Supplementary Figure 5. Example of legitimate merger from Mac1, wherein the acenaphthylene core is chemically sound, but for which no analogues are present in make-on-demand space.
